# Self-Attention-Based Deep Convolution LSTM Framework for Sensor-Based Badminton Activity Recognition

**DOI:** 10.3390/s23208373

**Published:** 2023-10-10

**Authors:** Jingyang Deng, Shuyi Zhang, Jinwen Ma

**Affiliations:** School of Mathematical Sciences and LMAM, Peking University, Beijing 100871, China; jingyang@stu.pku.edu.cn (J.D.); zzssyy@math.pku.edu.cn (S.Z.)

**Keywords:** badminton activity recognition, deep learning, Long Short-Term Memory (LSTM), self-attention

## Abstract

Sensor-based human activity recognition aims to classify human activities or behaviors according to the data from wearable or embedded sensors, leading to a new direction in the field of Artificial Intelligence. When the activities become high-level and sophisticated, such as in the multiple technical skills of playing badminton, it is usually a challenging task due to the difficulty of feature extraction from the sensor data. As a kind of end-to-end approach, deep neural networks have the capacity of automatic feature learning and extracting. However, most current studies on sensor-based badminton activity recognition adopt CNN-based architectures, which lack the ability of capturing temporal information and global signal comprehension. To overcome these shortcomings, we propose a deep learning framework which combines the convolutional layers, LSTM structure, and self-attention mechanism together. Specifically, this framework can automatically extract the local features of the sensor signals in time domain, take the LSTM structure for processing the badminton activity data, and focus attention on the information that is essential to the badminton activity recognition task. It is demonstrated by the experimental results on an actual badminton single sensor dataset that our proposed framework has obtained a badminton activity recognition (37 classes) accuracy of 97.83%, which outperforms the existing methods, and also has the advantages of lower training time and faster convergence.

## 1. Introduction

Human Activity Recognition (HAR) aims to use sensor-based or other related data to classify and recognize human activities. In fact, HAR has played a key role in many practical applications, such as in living behavior analysis [1], healthcare [2], gesture recognition [3], and sport activity recognition [4], etc. Therefore, HAR has attracted extensive attention from both academic and technical communities owing to its practicality.

According to different methods of data collection, research on HAR can be roughly categorized into two categories: video-based and sensor-based HARs. Obviously, the former is based on the image and video data collected using optical sensors (such as cameras), while the latter is based on the raw data from wearable or environmental sensors (such as accelerometers, gyroscopes, and magnetometers). In the past decade, most of the HAR research was video-based, since video data are easier to collect and more affordable than sensor data [5]. However, the high computational complexity of analyzing and processing 3D video data makes it difficult to establish a real-time effective recognition system. Moreover, video-based HAR is more sensitive to camera settings, such as placement, viewing angle, and focal length, which inevitably decreases the recognition accuracy [6]. With the quick development of sensor technology, sensor-based methods gradually have the advantages of both the feasibility and reliability of HAR tasks and also have good privacy protection [7]. In the existing sensor-based HAR research, as well as the related public datasets, researchers generally focus on basic behaviors such as walking, standing, sitting, going up and down stairs, and biking [8,9,10,11], etc. However, there are relatively few investigations on high-level fine movements, such as different racket swings when playing badminton [12,13].

In the sensor-based HAR, a series of conventional machine learning models and algorithms have been applied and tested, such as Support Vector Machine (SVM) [14], K-Nearest Neighbours (KNN) [15], and Random Forest (RF) [16], etc. In fact, all of them can achieve good results in certain cases, especially for simple activity recognition such as walking and running. In these settings, conventional machine learning approaches have shown competitive results. However, those conventional machine learning algorithms rely on the heuristic manual extraction of features, and the quality of feature extraction directly affects the recognition accuracy. In addition, these hand-crafted features are feasible only for simple activity recognition. When facing more sophisticated problems, they cannot perform so well in general since the effective features are very difficult to extract in these situations [7].

In order to overcome the above weaknesses of conventional machine learning algorithms, deep learning neural networks have been adopted for HAR. The deep learning methods can extract effective features automatically by implementing a succession of nonlinear transformations, which shows the promise that a proper deep learning method may reach a higher upper bound of accuracy in a complex recognition task, compared with conventional machine learning methods. In the training process of deep learning methods, the stochastic gradient descent algorithm is applied to learn the weights of the neural network from the labelled sample data. In fact, Convolutional Neural Network (CNN) and Recurrent Neural Network (RNN) are the two most popular deep neural networks applied in the research of HAR. Many investigations and applications have shown that CNN is effective for automatically extracting features from sensor data, while RNN is effective for mining the temporal information of sensor data [17].

Recently, the hybrid deep learning models combining CNN and RNN together [9,17] have achieved better performance than the single CNN models because they have a recurrent layer to take the temporal relationship of sensor data into account. In fact, if the recurrent layer and the convolutional layer combine together, the temporal features can be extracted from different sensor modalities. Moreover, by the introduction of an attention mechanism, a series of attention-based methods have emerged, such as adding an attention layer on the recurrent layer [10,18] and designing attention among different sensor modalities [10]. These attention-based deep learning models further enhance the accuracy rate of HAR on many public datasets.

As a relatively new network structure, the self-attention mechanism was proposed by Google in the machine translation model Transformer [19] in 2017. It firstly showed its power in natural language processing and has made outstanding achievements in many machine learning tasks. This special structure can be adopted into a general neural network architecture to capture the context information in the sequence from multiple aspects by calculating the distribution of the weights in the temporal domain. In this way, the self-attention-based model can adjust the focus of output layers on the crucial part of the result, thereby minimizing the impact of noise information on the output result [11].

As for badminton activity recognition, this is a key technology for modern intelligent sports [13]. Several applications, such as a badminton AI coach and AI rating system, can be developed based on the recognition results, which is beneficial for athletes and amateurs. To our best knowledge, most research on badminton activity recognition adopts conventional machine learning algorithms and CNN-based architectures. Among them, some CNN-based approaches focus on mining and utilizing the relationship among different activity classes to boost the recognition performance, such as in references [12,13]. However, conventional machine learning algorithms are strongly dependent on the quality of heuristic feature extraction that usually requires specialized domain knowledge, while the CNN architecture lacks the ability to capture temporal features and global sensor signal comprehension. Moreover, according to the direct experiments on our specialized Badminton Single-Sensor (BSS) dataset, current deep learning models are still far from fitting this collected dataset. In order to enhance the expression ability of the model, we add the recurrent structure for the temporal dependency of the sensor signals and adopt the self-attention mechanism to construct a deep learning neural network framework, called SADeepConvLSTM, for both high recognition performance and lightweight deployment. We conduct experiments on the BSS dataset, with each sample containing sensor data of tri-axis accelerations and attitude angles, to validate the effectiveness of our framework. In summary, we make the following contributions:

1. To overcome the shortcomings of the current methods for badminton activity recognition, we propose a new framework SADeepConvLSTM which combines the convolutional, recurrent, and self-attention layers together for synthetically improving the recognition performance. Such a design strategy has never been explored by previous work on badminton activity recognition.
2. The adopted LSTM and self-attention layers in SADeepConvLSTM are able to effectively extract temporal features from the sensor signals and suppress the noise interference, which leads to the acceleration of the recognition process and an increase in the accuracy and macro F1-score at the same time.
3. Compared with the existing popular deep learning models for badminton activity recognition on the specialized BSS dataset, SADeepConvLSTM obtains the best recognition accuracy. Moreover, it also has the advantages of lower training time and faster convergence.

The rest of this paper is organized as follows. We review the related work of sensor-based badminton activity recognition in Section 2. Our proposed deep learning neural network framework and learning algorithms are presented in Section 3. The experimental results are summarized in Section 4. Finally, we make a brief conclusion in Section 5.

## 2. Related Work

The existing badminton activity recognition approaches can be divided into two categories: conventional machine learning methods and deep learning methods. We review and summarize the badminton activity recognition research of these two categories separately as follows.

### 2.1. Conventional Machine Learning

Conventional machine learning approaches rely on classic hand-crafted features, such as mean, variance, maximum, difference, and Fast Fourier Transform (FFT) coefficients [20]. As for a HAR task, these extracted features are input into a supervised machine learning algorithm like SVM or KNN. In 2016, Anik et al. [15] designed a complete system, from data collection to recognition, for badminton games. SVM and KNN classifiers were utilized to recognize several predefined badminton activities according to the accelerometer and gyroscope data with a fast and low-cost solution. Their experimental results demonstrated that the SVM can obtain a satisfactory recognition rate of 88.9%. In fact, the other machine learning algorithms were also utilized and tested for badminton activity recognition. For example, Wang et al. [21] proposed a two-layer Hidden Markov Model (HMM) to classify badminton strokes into fourteen categories. The sensor data of the accelerometer and gyroscope were also collected in this study. The experimental results showed that the two-layer HMM classification algorithm can achieve the best performance in terms of recognition accuracy and recognition time. Ma et al. [22] further proposed a frequency-weighted training method taking inputs of a single accelerometer to improve the performance of HMM on badminton hitting action recognition.

The above conventional machine learning algorithms can perform well in certain simple or specific badminton action recognition tasks. However, when employing these algorithms, feature extraction is an indispensable step and requires the intervention of professional knowledge [7], which becomes more complicated and difficult for the general and complicated badminton action recognition tasks.

### 2.2. Deep Learning

Deep learning approaches resort to deep neural networks to perform automatic feature extraction and classification with a promising solution for HAR. So far, most existing research on badminton activity recognition adopted CNN models for effectively extracting local features from the input data. Wang et al. [6] proposed the AFEB-AlexNet framework to relieve the problem of data dislocation and enhance the performance of CNN for badminton action recognition to an accuracy of 98.65% in a ten-class-classification task. Steels et al. [23] also investigated the badminton activity recognition using the accelerometer data and the experimental results showed that their CNN model can recognize nine activities with an accuracy of 86% when using a sampling frequency of 50 Hz. As the accelerometer data and gyroscope data were combined together, the recognition accuracy could increase to 99%. Anand et al. [24] designed a sports analytics system that efficiently distinguishes the intricacies of players’ hand movements for several sports like tennis, badminton, and squash. Both CNN and Bi-directional LSTM (BiLSTM) were used for the shot classification, but BiLSTM obtained a slightly higher accuracy than CNN. The accelerometer and gyroscope were utilized by all the above studies to perform badminton activity recognition tasks.

Although there are relatively few approaches using RNN to recognize badminton activities, the prospects of RNN have been deeply explored in general HAR tasks because the structure of RNN is more suitable for extracting sequential features from the sensor temporal data. The Long Short-Term Memory (LSTM) networks and the Gated Recurrent Unit (GRU) networks are two of the most widely used RNN networks in HAR. Hammerla et al. [25] examined the performance of DNN, CNN, and several LSTM variants on three public datasets. The experimental results discovered that CNN and LSTM have their own advantages on different datasets and in various recognition metrics, but both are better than DNN. Later, Guan et al. [26] proposed an ensemble learning algorithm for multiple LSTM networks, which was demonstrated to have better recognition performance than a single LSTM network on the standard dataset.

Moreover, the CNN-RNN hybrid model has shown promising results in HAR tasks [9,17]. Ordóñez et al. [17] proposed the DeepConvLSTM model based on 1D convolution in the time domain and the LSTM recurrent layer. This model defeated a series of machine learning algorithms submitted on the OPPORTUNITY challenge, including SVM, C4.5 DT, and KNN. Moreover, the experimental results showed that adding some recurrent layers after the last convolutional layer can lead to better performance. In contrast to inputting raw sensor signals to the model, Yao et al. [9] proposed a more complex DeepSense model based on CNN-RNN, which solves the problems of classification and regression at the same time. Specifically, the signal was divided into several small segments, and each of them was then implemented using Fast Fourier Transform. Next, the derived amplitudes and phases were fed into different CNN sub-networks to extract and fuse multi-modal features. Finally, a two-layer GRU and a fully connected layer were used to capture the temporal information of these segments and generate the output.

The adoption of the attention mechanism has led to a series of variants of the existing models. Murahari et al. [18] added an attention module to DeepConvLSTM (referred to as DeepConvLSTM_Att), which could improve the accuracy of recognition. Based on DeepSense, Ma et al. [10] designed an attention mechanism to the data of different modalities and the output of the last GRU layer to establish the AttnSense model.

The emergence of the self-attention mechanism resulted in a breakthrough in the field of NLP [19]. By applying multiple self-attentive blocks and attention modules, we can easily construct an attention-based neural network architecture without convolutional and recurrent layers. Inspired by the aforementioned, Mahmud et al. [27] compared the multi-dimensional activity data at a frame to a word vector representation in the sentence and proposed an analogy model (referred to as SelfAttnNet) with the macro F1-score being higher than that of the other existing models. Betancourt et al. [11] and Yao et al. [28] further added a self-attention module to LSTM and DeepSense, respectively, to improve the recognition accuracy.

## 3. Materials and Methods

This section begins by introducing the experimental dataset used in this study. Subsequently, we describe our proposed deep learning neural network framework—the Self-Attention-Based Deep Convolutional LSTM (SADeepConvLSTM), as well as its learning algorithms.

### 3.1. Badminton Single-Sensor (BSS) Dataset

**Figure 1:**
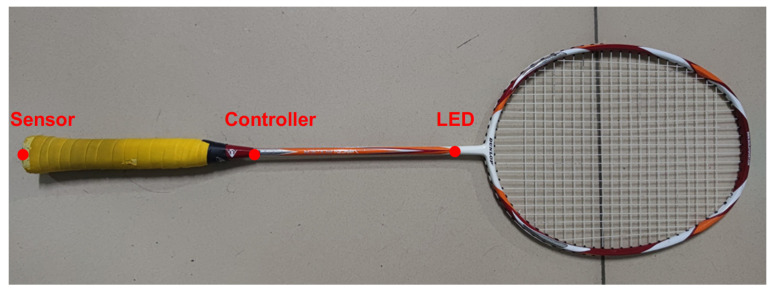
Sensor deployment of the badminton racket.

Our experiments are conducted on a specialized Badminton Single-Sensor (BSS) dataset which had been collected by the China Institute of Sport Science (CISS). The dataset was generated to analyze various standard swing motions and, finally, build an AI coach system. Under the guidance of sports experts, we divided all professional badminton swing movements into 37 fine-grained classes to conduct refined recognition, including forehand high serve, backhand hook diagonal, overhead smash, etc. During the process of data collection, two professional athletes were asked to swing according to the standard technical movement to eliminate differences between the subjects. A total of 4801 samples were collected in our BSS dataset (about 130 samples per class on average), with each class containing at least 100 samples to ensure the dataset is relatively balanced. As shown in Figure 1, to eliminate the impact of the deployed equipment on the athlete’s movements, a small specially made sensor is inset into the handle bottom of the badminton racket to sample the signal at a frequency of 200 Hz. The features of interest mainly fall into two categories: (1) the three-axis accelerations of the sensor in *x*, *y*, and *z* axes, measured using an accelerometer; (2) the attitude angles of the human joint relative to the geodetic coordinate system, namely pitch angle θ, yaw angle ψ, and roll angle ϕ, evaluated using a gyroscope. In our experiments, we treat raw inputs as six-dimensional time series.

For each sample, the number of frames depends on when the sensor turns on and off. The whole collecting process is artificially controlled and hence highly subjective. Therefore, for the convenience of training and testing, we merely consider 300 frames of each sample around the time at which the athlete is swinging the racket. Specifically, we firstly find out the maximum point of the resultant acceleration to determine when the athlete exerts the force, and then unify the length of each sample to 300 and carry out the corresponding processing to truncate the redundant frames or pad the shortage data with zero. Then, we randomly select 70% of all samples as the training set, while the remaining 30% form the testing set to evaluate the recognition accuracy.

### 3.2. Proposed Framework and Learning Algorithms

For a multi-class badminton activity recognition problem, we let $A=\{A_{1},A_{2},\ldots ,A_{N}\}$ be the set of activities given in advance, where $A_{i}\;\left(i=1,2,\ldots ,N\right)$ are *N* different activity categories or classes, and let X be the set of all possible sensor data collected. A deep learning approach tries to establish a neural network model $F_{\omega }:X\to P\subset R^{N}$ directly to recognize *N* different activities, where ω denotes all the parameters of the model $F_{\omega }$, and P is the set of all possible probability distributions over the *N* activity categories (or classes). For the parameter learning, we use a loss function L:P×A→R to measure the gap between the predicted distribution and the ground truth, then find out the optimal solutions of the parameters ω via solving the following end-to-end optimization problem:

(1) $$\underset{\omega }{\arg\min}J\left(\omega \right)=\frac{1}{M}\sum_{i=1}^{M}L\left(F_{\omega }\left(X^{\left(i\right)}\right),A^{\left(i\right)}\right),$$

where *M* is the number of samples in the training set, $X^{\left(i\right)}\in X$ the *i*-th sample in the training set, and $A^{\left(i\right)}\in A$ denotes the activity category ground truth of $X^{\left(i\right)}$. When performing the recognition task for a given sample X∈X, we can select its activity category, i.e., the class, as the number of the unit with the largest predicted probability in the soft-max output layer; that is, we compute $k={\arg\max}_{i}{\left(F_{\omega }\left(X\right)\right)}_{i}$, $A_{k}\in A$ to be the activity category.

In the following subsections, we begin to briefly introduce the DeepConvLSTM model [17], and then describe our proposed framework SADeepConvLSTM and its self-attention-based network structure, as well as the related learning algorithms.

#### 3.2.1. DeepConvLSTM

**Figure 2:**
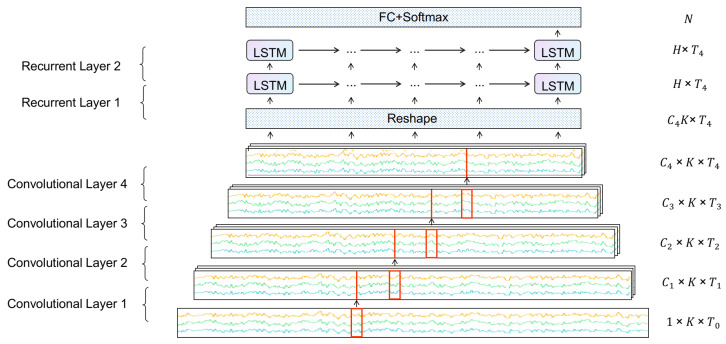
Structure of the DeepConvLSTM model.

The DeepConvLSTM model consists of three components: convolutional layers, recurrent layers, and fully connected layers. Its structure is shown in Figure 2, where $C_{i}$ is the number of channels of the *i*-th convolutional layer, $T_{i}$ is the number of time-steps of the *i*-th convolutional layer’s output, *K* is the number of signal series, *H* is the dimension of the LSTM hidden layer, and *N* is the number of activity classes.

The direction of the time axis in Figure 2 is from left to right. The input data are sequentially processed through 4 convolutional layers to extract local temporal features. The red boxes in convolutional layers denote convolutional kernels, the following two recurrent layers perform nonlinear transformations to the processed data, and then the uppermost fully connected layer and softmax layer perform activity classification and output the recognition result. The right column of the figure shows the dimensions of the data in each layer. We further discuss the details of each layer as follows.

(a) Convolutional Layer. The first to fourth layers of the DeepConvLSTM model are all 1D convolutional layers. Different channels are convolved separately with the same kernel. Letting the data of the *k*-th sensor at time *t* in the *i*-th layer, *j*-th channel be $x_{jk}^{\left(i\right)}\left(t\right)$, we denote the bias of the *j*-th kernel in the *i*-th layer as $b_{j}^{\left(i\right)}$, and denote the value of its τ-th parameter on the channel *c* be $K_{cj}^{\left(i\right)}\left(\tau \right)$. So, the operation of one-dimensional convolution can be mathematically expressed by
  (2) $$x_{jk}^{\left(i+1\right)}\left(t\right)=\sigma \left(b_{j}^{\left(i\right)}+\sum_{c=1}^{C_{i}}\sum_{\tau =1}^{S_{i}}K_{cj}^{\left(i\right)}\left(\tau \right)x_{ck}^{\left(i\right)}\left(t-\tau \right)\right),$$
  where $C_{i}$ denotes the number of convolution kernels in the *i*-th layer, and $S_{i}$ denotes the size of the kernel in the *i*-th layer. In fact, σ is a nonlinear activation function, and is usually taken as the ReLU function; that is, σ(x)=max(x,0).
(b) Recurrent Layer. The fifth and sixth layers of the DeepConvLSTM model are recurrent layers, and their structures are adopted as the classical LSTM network that contains input, output, and forgetting gates:
  (3) $$\begin{array}{cc} f_{t} & =\sigma (W_{f}\cdot \left[h_{t-1},x_{t}\right]+b_{f})\\ i_{t} & =\sigma (W_{i}\cdot \left[h_{t-1},x_{t}\right]+b_{i})\\ {\tilde{C}}_{t} & =\tanh(W_{C}\cdot \left[h_{t-1},x_{t}\right]+b_{C})\\ C_{t} & =f_{t}*C_{t-1}+i_{t}*{\tilde{C}}_{t}\\ o_{t} & =\sigma (W_{o}\cdot \left[h_{t-1},x_{t}\right]+b_{o})\\ h_{t} & =o_{t}*\tanh\left(C_{t}\right), \end{array}$$
  where $x_{t}$ denotes the input data, and $C_{t}$ and $h_{t}$ denote the hidden state and the output of the *t*-th cell, respectively. σ is a nonlinear activation function, and $W_{f}$, $b_{f}$, $W_{i}$, $b_{i}$, $W_{C}$, $b_{C}$, $W_{o}$, and $b_{o}$ are learnable parameters.
(c) Fully Connected Layer and Softmax Layer. The output of the last time-step of the sixth layer is then input into the fully connected layer, and the obtained *N* dimension vector is then input to the last Softmax layer to get the corresponding probability distribution P=F(X)∈P. Letting $x={\left\{x_{i}\right\}}_{i=1}^{N}\in R^{N}$, the output of the Softmax layer can be defined by
  (4) $$\mathrm{Softmax}\left(x\right)=\frac{1}{Z(x)}{\left\{e^{x_{i}}\right\}}_{i=1}^{N},$$
  where
  (5) $$Z\left(x\right)=\sum_{j=1}^{N}e^{x_{j}}$$
  is the normalized coefficient.

#### 3.2.2. Self-Attention-Based Deep Convolutional LSTM (SADeepConvLSTM)

**Figure 3:**
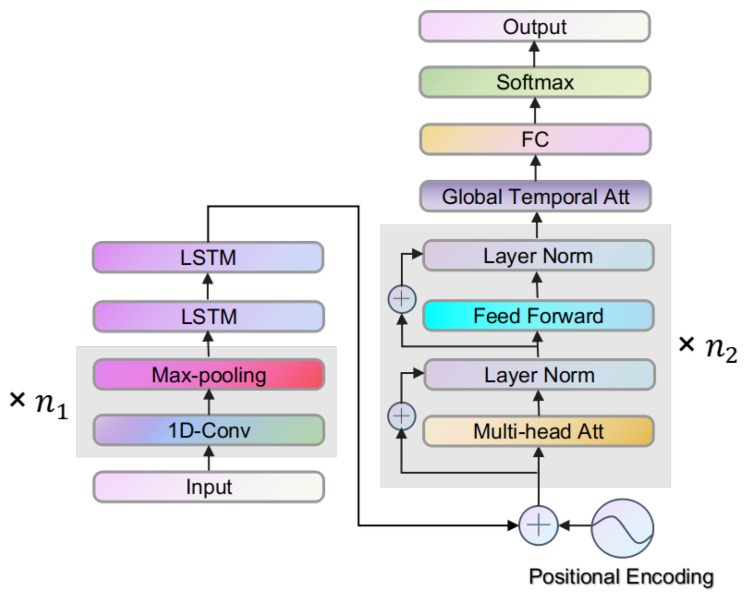
Structure of the SADeepConvLSTM Framework.

In order to improve the accuracy of badminton activity recognition, we adopt the self-attention mechanism into the DeepConvLSTM model to establish the self-attention-based deep convolutional LSTM framework, i.e., the SADeepConvLSTM framework. In fact, the self-attention mechanism enables the framework to capture the crucial context information in the sequence and the crucial relationship between the features of different time-steps. Figure 3 shows our proposed SADeepConvLSTM framework, where the modules with the gray background can repeat several times in succession. The left half of the structure follows the DeepConvLSTM framework with maximum pooling layers, while the right half is the structure related to the self-attention mechanism. We now describe and analyze the structure and learning algorithms of the SADeepConvLSTM framework as follows.

The fast development of sensor technology greatly promotes the sampling frequencies, enabling the sensors to collect hundreds of frames of data for an action in a few seconds. In this case, it takes a lot of computing time and resources to pass the data into the original DeepConvLSTM model to perform the activity recognition. Although LSTM can relieve the gradient disappearance encountered by RNN to a certain extent, the effect of long-term memory cannot be so good when dealing with the problem of super-long sequences. Considering the above two points, we add a max pooling layer after each convolutional layer to reduce the number of time-steps of the recurrent layer.

Each component of the framework is described as follows.

(a) Positional Encoding. Positional Encoding aims to add temporal positional information to the hidden features before feeding them into the self-attention module. In fact, the self-attention module given and discussed in the following Section 3.2.2 does not consider positional information; that is, the same embedding vector at different time-steps generates the same attention values.
  We adopt the position encoding method suggested by Vaswani et al. [19] into our SADeepConvLSTM framework. By denoting the number of time-steps of the previous recurrent layer as *L*, and the dimension of each hidden feature as $d_{\mathrm{model}}$, the *k*-th components of the position encoding at the pos-th time-step can be calculated by
  (6) $${\mathrm{PE}}_{\left(\mathrm{pos},k\right)}=\left\{\begin{array}{cc} \sin\left(\frac{\mathrm{pos}}{10000^{2i/d_{\mathrm{model}}}}\right)\; & k=2i;\\ \cos\left(\frac{\mathrm{pos}}{10000^{2i/d_{\mathrm{model}}}}\right)\; & k=2i+1, \end{array}\right.$$
  where pos∈{0,1,…,L−1}, and $k\in \{0,1,\ldots ,d_{\mathrm{model}}-1\}$. For clarity, we denote the feature matrices before and after the positional embedding by $Y\in R^{L\times d_{\mathrm{model}}}$ and $\tilde{Y}\in R^{L\times d_{\mathrm{model}}}$, respectively.
(b) Self-Attention Module. There are two main components in the self-attention module: multi-head attention layer and feed-forward network. The attention mechanism computes the relative weights of the query vector $q_{i}$ by considering the dot product similarity between $q_{i}$ and the key vector $k_{j}$ and then appending the weights to the value vector $v_{j}$ and summing up to obtain the attention value of $q_{i}$. Mathematically, letting $q_{i}$, $k_{j}$, and $v_{j}$ be row vectors, $K={\left[k_{1}^{T}\;k_{2}^{T}\;\cdots \;k_{n}^{T}\right]}^{T}$, $V={\left[v_{1}^{T}\;v_{2}^{T}\;\cdots \;v_{n}^{T}\right]}^{T}$, we then have
  (7) $$\mathrm{Attention}\left(q_{i},K,V\right)=\sum_{j=1}^{n}w_{j}v_{j},$$
  where
  (8) $$w=\mathrm{Softmax}\left(q_{i}\cdot k_{1}/{\sqrt{d}}_{k},\ldots ,q_{i}\cdot k_{n}/{\sqrt{d}}_{k}\right),$$
  $d_{k}$ is the dimension of $q_{i}$ and $k_{j}$ and is a constant zoom factor. The matrix form of the above equation can be written as
  (9) $$\mathrm{Attention}\left(q_{i},K,V\right)=\mathrm{Softmax}\left(\frac{q_{i}K^{T}}{{\sqrt{d}}_{k}}\right)V.$$
  Letting $Q={\left[q_{1}^{T}\;q_{2}^{T}\;\cdots \;q_{n}^{T}\right]}^{T}$, since Equation (8) holds for any *i*, we thus have
  (10) $$Z≜\mathrm{Attention}\left(Q,K,V\right)=\mathrm{Softmax}\left(\frac{QK^{T}}{{\sqrt{d}}_{k}}\right)V\in R^{L\times d_{v}},$$
  where Softmax can be considered as being computed by row.
  In the self-attention mechanism, the aforementioned *Q*, *K*, and *V* are all generated by applying nonlinear transformations to the input $\tilde{Y}$:
  (11) $$Q=\tilde{Y}W_{Q},\;K=\tilde{Y}W_{K},\;V=\tilde{Y}W_{V},$$
  where $W≜\{W_{Q},W_{K},W_{V}\}$ are learnable parameters. $W_{Q}$, $W_{K}\in R^{d_{\mathrm{model}}\times d_{k}}$, $W_{V}\in R^{d_{\mathrm{model}}\times d_{v}}$, where $d_{k}$ is the dimension of $q_{i}$ and $k_{i}$, while $d_{v}$ is the dimension of $v_{i}$. By introducing the self-attention mechanism, we can capture the crucial context information during the whole process of an action.
  In our SADeepConvLSTM framework, we adopt the multi-head attention [19] to extract the features in multiple aspects. In other words, we use *n* different sets of learnable parameters $W^{\left(1\right)},W^{\left(2\right)},\ldots ,W^{\left(n\right)}$ to generate different $Q^{\left(i\right)}$, $K^{\left(i\right)}$, and $V^{\left(i\right)}$ to compute the attention values $Z^{\left(i\right)}$ and concatenate them together. If $nd_{v}\neq d_{\mathrm{model}}$, we use the learnable parameters $W_{O}\in R^{nd_{v}\times d_{\mathrm{model}}}$ to convert the input into the original dimension:
  (12) $$Z_{\mathrm{mha}}=\left(Z^{\left(1\right)}\;Z^{\left(2\right)}\;\cdots \;Z^{\left(n\right)}\right)W_{O}.$$
  Otherwise, we directly let
  (13) $$Z_{\mathrm{mha}}=\left(Z^{\left(1\right)}\;Z^{\left(2\right)}\;\cdots \;Z^{\left(n\right)}\right).$$
  Subsequently, the resulting $Z_{\mathrm{mha}}$ is inputted into a feed-forward network consisting of 2 fully connected layers to implement the nonlinear transformations. In this module, we use the residual connections and layer normalization for both the multi-head attention and the feed-forward network.
(c) Global Temporal Attention. We use the learnable parameters to evaluate the relative importance of the feature representation obtained by the last self-attention module at each time-step:
  (14) $$\begin{array}{cc} u_{t} & =\tanh\left(W_{\omega }h_{t}+b_{\omega }\right)\\ \alpha_{t} & =\frac{\exp\left(u_{t}\cdot u_{\omega }\right)}{\sum_{t}\exp\left(u_{t}\cdot u_{\omega }\right)}. \end{array}$$
  where $h_{t}\in R^{d_{\mathrm{model}}}$ denotes the feature representation at time *t*. $W_{\omega }\in R^{d_{\mathrm{model}}\times d_{\mathrm{model}}}$, $b_{\omega }\in R^{d_{\mathrm{model}}}$, and $u_{\omega }\in R^{d_{\mathrm{model}}}$ are learnable parameters. Finally, we get the feature vector *h* by computing the weighted average of $h_{t}$:
  (15) $$h=\sum_{t}\alpha_{t}h_{t}.$$

#### 3.2.3. Training Process

For the parameter learning during the training process, we adopt the following cross entropy loss function:

(16) $$L\left(P,A\right)=-\sum_{i=1}^{N}q_{i}\log p_{i}$$

to measure the similarity between the predicted activity probability distribution and the ground truth, where $P=\{p_{1},p_{2},\ldots ,p_{N}\}$ and $Q=\{q_{1},q_{2},\ldots ,q_{N}\}$ are the predicted and true probability distributions over the badminton activity set *A*.

Moreover, to enhance the generalization ability of the framework, we even add a L-2 regularization term to the loss function J(ω) and get the new loss function as follows:

(17) $$\tilde{J}\left(\omega \right)=\sum_{i=1}^{M}L\left(F_{\omega }\left(X^{\left(i\right)}\right),A^{\left(i\right)}\right)+\frac{\lambda }{2}{∥\omega ∥}_{2}^{2}.$$

Furthermore, the dropout mechanism is also utilized between the layers to prevent over-fitting.

## 4. Experimental Results

In this section, several experiments are conducted to demonstrate our proposed SADeepConvLSTM framework and learning algorithms on our specialized BSS dataset. Moreover, it is compared with typical conventional machine learning algorithms and state-of-the-art deep learning models.

### 4.1. Evaluation Metrics

In the experiments, we use both the recognition accuracy rate and macro F1-score to measure the performance of each model on the badminton activity recognition task. The recognition accuracy rate is defined as the proportion of correctly recognized samples among all samples, while the macro F1-score is computed by

(18) $$\mathrm{Macro}\;F1\;\mathrm{Score}=\frac{1}{C}\sum_{i=1}^{C}\frac{2\times {\mathrm{Precision}}_{i}\times {\mathrm{Recall}}_{i}}{{\mathrm{Precision}}_{i}+{\mathrm{Recall}}_{i}},$$

where C=37 is the number of activity classes.

### 4.2. Experimental Settings

In practice, the sampling data are noisy according to various reasons, especially the interference from the environment. In order to alleviate this problem, we use a low-pass filter, the Butterworth filter, to separate the noise with high frequency from the signal. As for feature extraction in this specific situation, we adopt the sliding window method that is actually a common method for extracting features in HAR [29]. It considers each small window $\left\{X_{i},X_{i+1},\ldots ,X_{i+t-1}\right\}$ with the duration of t(t≤T) of the sample data, and computes the statistical and physical features from its elements. After referring to the previous works [20,29], we select the effective features such as mean, median, mean absolute deviation, correlation coefficients, mean resultant acceleration, difference, and peak point location. We set the width of the sliding window *t* to 10 and the stride to 1 when performing the feature extraction.

For all the deep learning models, we use the Adam optimization algorithm to train the model. The initial learning rate is set to $10^{-3}$ with a decay rate of 0.1 per one hundred epochs. Ten repeated random experiments are conducted to reduce the error caused by randomness in the training process. We set the regularizer λ to be $10^{-5}$, the batch size to be 128, the dropout rate be 0.5, and the $\left(\beta_{1},\beta_{2}\right)$ in the Adam optimization algorithm to be $\left(0.9,0.999\right)$.

Our SADeepConvLSTM framework follows the settings of the DeepConvLSTM model, and takes the number of convolution-pooling modules $n_{1}=4$, 1D convolution kernel size $S_{1}=S_{2}=S_{3}=S_{4}=5$, and the number of channels $C_{1}=C_{2}=C_{3}=C_{4}=64$. The pooling kernel size is set to 2, and the dimensions of both the LSTM and self-attention hidden layer are set to 128, i.e., $H=d_{\mathrm{model}}=128$. The number of attention heads is set to 4, i.e., n=4. The dimensions of $q_{i}$, $k_{i}$, and $v_{i}$ are all set to 32, i.e., $d_{k}=d_{v}=32$. In addition, due to the residual connection structure, the self-attention module is usually stacked multiple times in the previous works (see, e.g., references [11,19,27]). However, considering that our system may be deployed on small mobile devices, the network structure is designed as simply as possible so that we set $n_{2}=2$. To further validate the effectiveness of our SADeepConvLSTM, we also relax this limitation and conduct experiments under $n_{2}=4$.

For comparison, we also train the other state-of-the-art deep learning models such as LSTM, BiLSTM, LSTM_Attention, DeepConvLSTM, DeepConvLSTM_Att, DeepSense, AttnSense, and SelfAttnNet. In all the models, we limit the dimension of a hidden layer to 128.

We further compare our SADeepConvLSTM framework with two typical conventional machine learning models: SVM and RF. For the SVM model, we use the Bayesian Optimization Searching to find out the best hyperparameters γ and *C* in each case, and use *K*-fold cross-validation on the training set to finalize the optimal hyperparameters, which is actually implemented by maximizing the following average macro F1-score:

(19) $$\mathrm{Average}\;\mathrm{Macro}\;F1\text{-}\mathrm{score}=\frac{1}{K}\sum_{k=1}^{K}\frac{1}{C}\sum_{i=1}^{C}\frac{2\times {\mathrm{Precision}}_{i}^{\left(k\right)}\times {\mathrm{Recall}}_{i}^{\left(k\right)}}{{\mathrm{Precision}}_{i}^{\left(k\right)}+{\mathrm{Recall}}_{i}^{\left(k\right)}},$$

where K=10 and C=37. ${\mathrm{Precision}}_{i}^{\left(k\right)}$ and ${\mathrm{Recall}}_{i}^{\left(k\right)}$ denote the precision and recall rates when the *k*-th fold of the data is set as the validation set, respectively.

For the RF algorithm, no more than the square root of the total number of features are randomly selected each time to train the CART decision tree model. We set the number of trees as much as the computer processing capacity and response time allows and finally train 10,000 weak classifiers for Bagging.

### 4.3. Results and Comparisons

We implement our SADeepConvLSTM framework as well as the other comparative models on the BSS dataset and the obtained average recognition accuracy rates and macro F1-scores are listed in Table 1.

**Figure 4:**
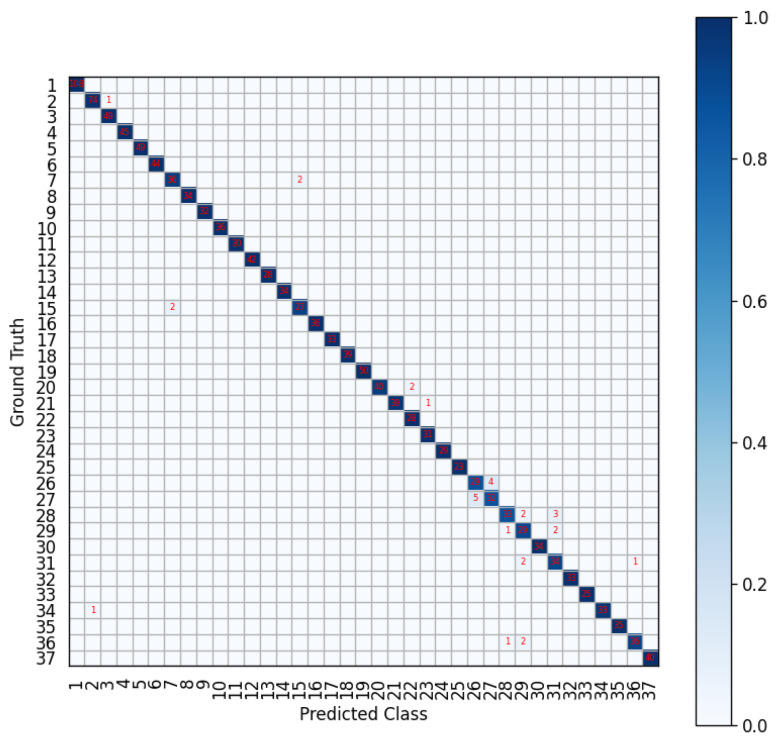
Recognition confusion matrix of the SADeepConvLSTM framework.

It can be seen from Table 1 that our SADeepConvLSTM framework achieves the best recognition result compared to other methods, with the average recognition accuracy rate of 97.83% and the average macro F1-score of 97.64%. Figure 4 further shows the confusion matrix of its recognition result, which states clearly that all the 37 classes are recognized very well. When we further relax the limitation on the model complexity, SADeepConvLSTM even yields a higher accuracy of 98.27% and macro F1-score of 98.17%. In general, our SADeepConvLSTM framework is remarkably better than other deep learning models.

From Table 1, we can also find out that the self-attention module really brings a significant improvement in the recognition accuracy rate (from 94.30% to 97.83%) and macro F1-score (from 93.98% to 97.64%) to the DeepConvLSTM model, and is better than the attention structure proposed by Murahari et al. [18] (see DeepConvLSTM_Att).

In addition, we also conduct an ablation study to analyze the effect of different layers in the SADeepConvLSTM framework. We removed all the max-pooling layers (see SADeepConvLSTM_NP) and all the convolution-pooling modules (see SADeepConvLSTM_NC), respectively, and neither of them can outperform the SADeepConvLSTM framework, which further shows the effectiveness of each component of our framework.

Compared with the SADeepConvLSTM framework, the SelfAttnNet model which is entirely based on the attention mechanism, lacks the local temporal features and long-term memory features extracted by the 1D convolutional layers and recurrent layers. It processes the input by assigning attention to different sensor modalities. Therefore, its recognition ability can be impaired when the dataset is relatively simple, and its performance on the BSS dataset is also inferior to the SADeepConvLSTM framework. The same explanation still applies to the AttnSense model, whose performance on the dataset is also affected and is worse than that of DeepSense, which relies on CNN for feature fusion. The basic models such as LSTM and BiLSTM are subject to their relatively simple structure and show poor results on the dataset. However, adding a global attention layer (see LSTM_Attention and BiLSTM_Attention) above the last recurrent layer can improve the model’s recognition ability to a great extent.

As for the two conventional machine learning models, SVM and RF, they both achieve an accuracy rate and macro F1-score of over 96%, and the RF algorithm achieves slightly better results than SVM with an accuracy rate of 96.53% and a macro F1-score of 96.27%. Therefore, those conventional machine learning models can still perform as well as some deep learning algorithms as long as the feature extraction is suitable. However, we should mention that numerous hand-crafted features were exhaustively attempted and a wide range of hyperparameters were carefully searched to ensure both SVM and RF achieve their best results. Without these efforts, the accuracy and macro F1-score of SVM are 94.38% and 94.27%, respectively. Meanwhile, RF reaches an accuracy of 94.66% and macro F1-score of 94.49% in this experiment setting. Although the SVM and RF outperform some deep learning methods, which is partly due to our intentional limitation on the complexity of deep models, our proposed SADeepConvLSTM framework is remarkably better than those conventional machine learning methods, reducing the recognition error rate by about 37.5% (from 3.47% for RF to 2.17%). When the limitation on model size is relaxed, we can reduce the recognition error by 50.1% (from 3.47% for RF to 1.73%).

**Figure 5:**
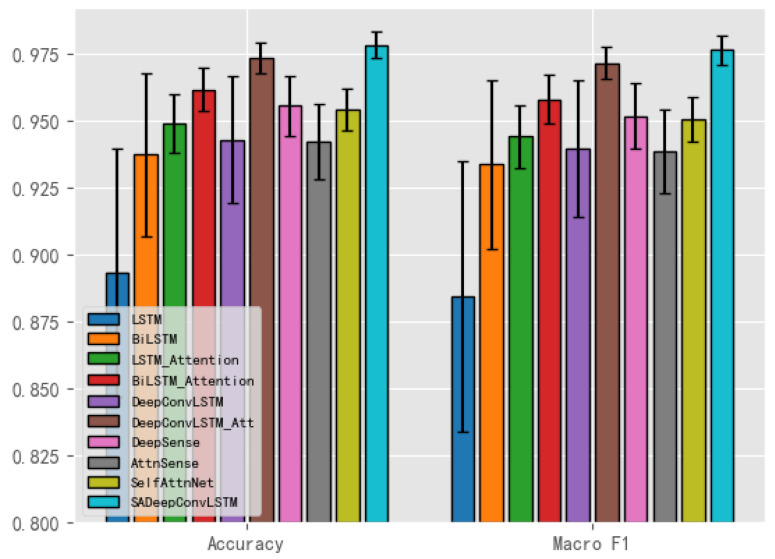
Barplot of the recognition results of the deep learning models.

Furthermore, the corresponding barplot of the experimental results is shown in Figure 5, where the error bars represent the confidence interval with the confidence level of 95%. The results of basic models such as LSTM and BiLSTM have shown wider fluctuation due to their simple structure and unstable training process, while the other models are relatively stable and show consistent results.

**Figure 6:**
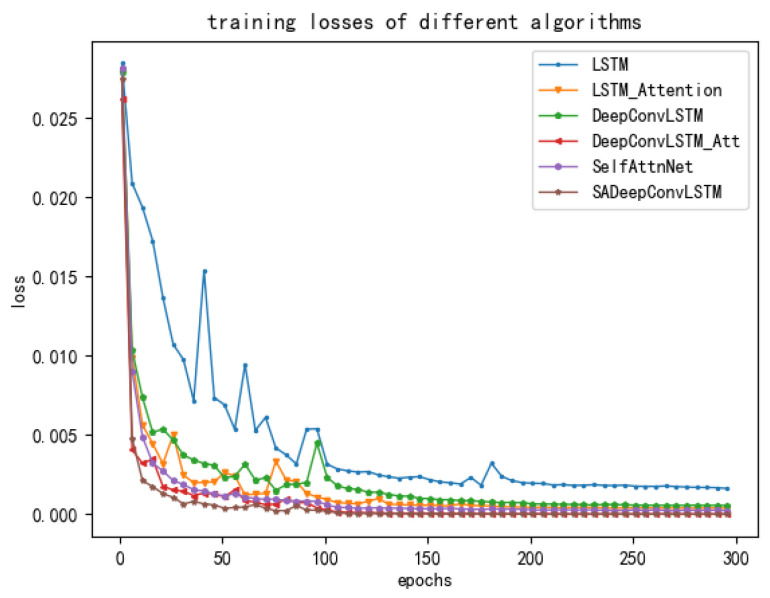
Sketches of the training losses of deep learning models with time.

**Figure 7:**
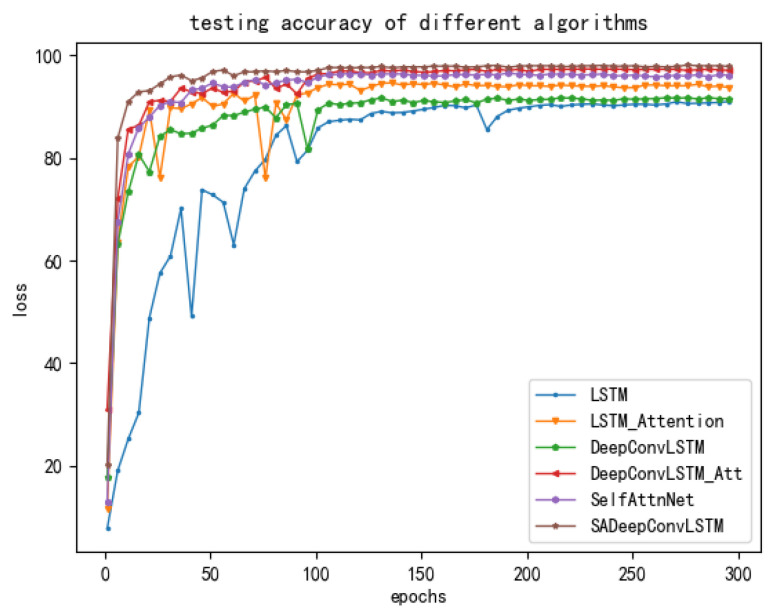
Sketches of the testing accuracy rates of deep learning models with time.

On the other hand, the changes in the loss and accuracy rate of the proposed and comparative models in the training are shown in Figure 6 and Figure 7, respectively. It can be found clearly in those two figures that our proposed SADeepConvLSTM framework converges fast and has a stable training process. On the contrary, the training process of the LSTM is unstable and shows a poor convergence rate.

Finally, we investigate the training times of different deep learning models for this badminton activity recognition task. Table 2 lists the training times of different deep learning models on the BSS dataset in the GPU computing environment. The SADeepConvLSTM framework reduces the number of time-steps in the recurrent layer due to the use of the max-pooling layer, and, therefore, its training time is relatively less than most of the other comparative deep learning models such as DeepConvLSTM. Although our SADeepConvLSTM adopts multiple types of structures, we keep the requirements for lightweight deployment in mind, and the model can train on a portable computer within about 7.2 min, and perform recognition tasks within merely $2\times 10^{-5}$ s for each sample, showing its lessened cost of computation.

## 5. Conclusions

We have established a self-attention-based CNN-LSTM hybrid deep learning framework, i.e., SADeepConvLSTM, for badminton activity recognition. Under the structure of the DeepConvLSTM model, we add the max-pooling layers after each convolutional layer to reduce the number of time steps in the recurrent layer to reduce its training time. Moreover, we introduce a self-attention mechanism to improve the recognition accuracy rate. It is demonstrated by a series of experiments on the BSS dataset that our proposed framework has obtained a recognition accuracy of 97.83% and outperforms the typical conventional machine learning algorithms and state-of-the-art deep learning models. Moreover, it even has the advantages of lower training time and faster convergence. However, our ultimate goals will not stop at badminton activity recognition. Several applications, such as an AI coach and AI rating system, can be developed based on our recognition results by analyzing differences between the motions of users and corresponding standard motions. Any improvement in the recognition accuracy may avoid potentially seriously misleading users in these applications. Therefore, our proposed method can play a key role in an integral sports AI system, owing to its high accuracy and low response time, which is beneficial for athletes and amateurs. Although this self-attention-based CNN-LSTM framework is directly designed for sensor-based badminton activity recognition, it can be easily applied and extended to other similar sensor-based HAR tasks. In more complex contexts, such as smart homes and smart cities, where the number of potential combinations of sensors is much higher than in a badminton scenario, the attention mechanism between different sensor modalities [10] may further enhance our framework without introducing high computational cost.

## Figures and Tables

**Table 1:** Metrics of the proposed and comparative models on the BSS dataset.

	Accuracy	Macro F1
SVM	96.39%	96.15%
RF	96.53%	96.27%
LSTM	89.33%	88.45%
BiLSTM	93.73%	93.38%
LSTM_Attention	94.89%	94.43%
BiLSTM_Attention	96.17%	95.81%
DeepConvLSTM [17]	94.30%	93.98%
DeepConvLSTM_Att [18]	97.34%	97.15%
DeepSense [9]	95.55%	95.18%
AttnSense [10]	94.23%	93.86%
SelfAttnNet [27]	95.43%	95.06%
SADeepConvLSTM_NP	97.04%	96.82%
SADeepConvLSTM_NC	97.24%	97.00%
SADeepConvLSTM	**97.83%**	**97.64%**
SADeepConvLSTM ${}^{*}$	**98.27%**	**98.17%**

* This is the SADeepConvLSTM model under $n_{2}=4$; Bold in the table represents the best result.

**Table 2:** Training times of the deep learning models (s).

	Training Time
LSTM	583
BiLSTM	1322
LSTM_Attention	633
BiLSTM_Attention	1461
DeepConvLSTM [17]	1548
DeepConvLSTM_Att [18]	1626
DeepSense [9]	2496
AttnSense [10]	**234**
SelfAttnNet [27]	2012
SADeepConvLSTM	433

Bold in the table represents the best result.

## Data Availability

The data presented in this study are available on request from the corresponding author. The data are not publicly available due to privacy.
